# Giant inguinoscrotal hernia containing intestinal segments and urinary bladder successfully repaired by simple hernioplasty technique: a case report

**DOI:** 10.1186/s13256-015-0759-5

**Published:** 2015-11-28

**Authors:** Mohamed Tarchouli, Moulay-Brahim Ratbi, Mohamed Bouzroud, Badr Aitidir, Abdelmounaim Ait-Ali, Ahmed Bounaim, Khalid Sair

**Affiliations:** Department of Digestive Surgery I, Faculty of Medicine and Pharmacy, Mohammed V Military Hospital, Mohammed V University, Rabat, Morocco

**Keywords:** Giant inguinoscrotal hernia, Lichtenstein repair, Urinary bladder

## Abstract

**Introduction:**

Giant inguinoscrotal hernias are extremely rare nowadays, but they may still be encountered after years or even decades of neglect. Such hernias containing both bowel loops and urinary bladder have not been reported in the medical literature to date, to the best of our knowledge.

**Case presentation:**

We report a case of a 65-year-old Moroccan man who presented with giant right-sided and long-standing inguinoscrotal hernia with compromised quality of life due to walking difficulties and sexual discomfort. Computed tomography revealed a voluminous hernia sac containing small and large bowel loops, greater omentum, and urinary bladder. Surgical repair was done through the classical inguinal incision using the Lichtenstein tension-free hernioplasty technique. No debulking or abdominal enlargement procedure had to be performed, apart from a partial omentectomy.

**Conclusions:**

Giant inguinoscrotal hernia containing intestinal segments and urinary bladder is a challenging surgical disease. A Lichtenstein tension-free technique seems to be the best surgical procedure for both the patient and the operating surgeon. It should be used whenever possible in such cases.

## Introduction

Although inguinal hernia is one of the most common surgical disease states encountered in medical practice, its treatment has evolved and has become easily accessible, especially in high-income countries. Thus, giant inguinal hernia has become extremely rare and appears only after years of neglect or lack of an accessible surgical facility. It is defined as an inguinal hernia that extends below the midpoint of the inner thigh in standing position [1]. Because of the physiological changes associated with the loss of domain, surgical repair is often challenging and may lead to potentially fatal complications. Numerous surgical techniques have been described, but none has been adopted as a standard procedure. Further, reduction of the hernia contents via an enlarging internal inguinal ring is difficult and scarcely reported in the medical literature.

In this report, we describe an unusual case of a giant inguinoscrotal hernia containing bowel loops and urinary bladder, which we managed through the classical inguinal incision using the Lichtenstein tension-free hernioplasty technique. We also highlight different problems relative to the management of this uncommon disease.

## Case presentation

**Fig. 1:**
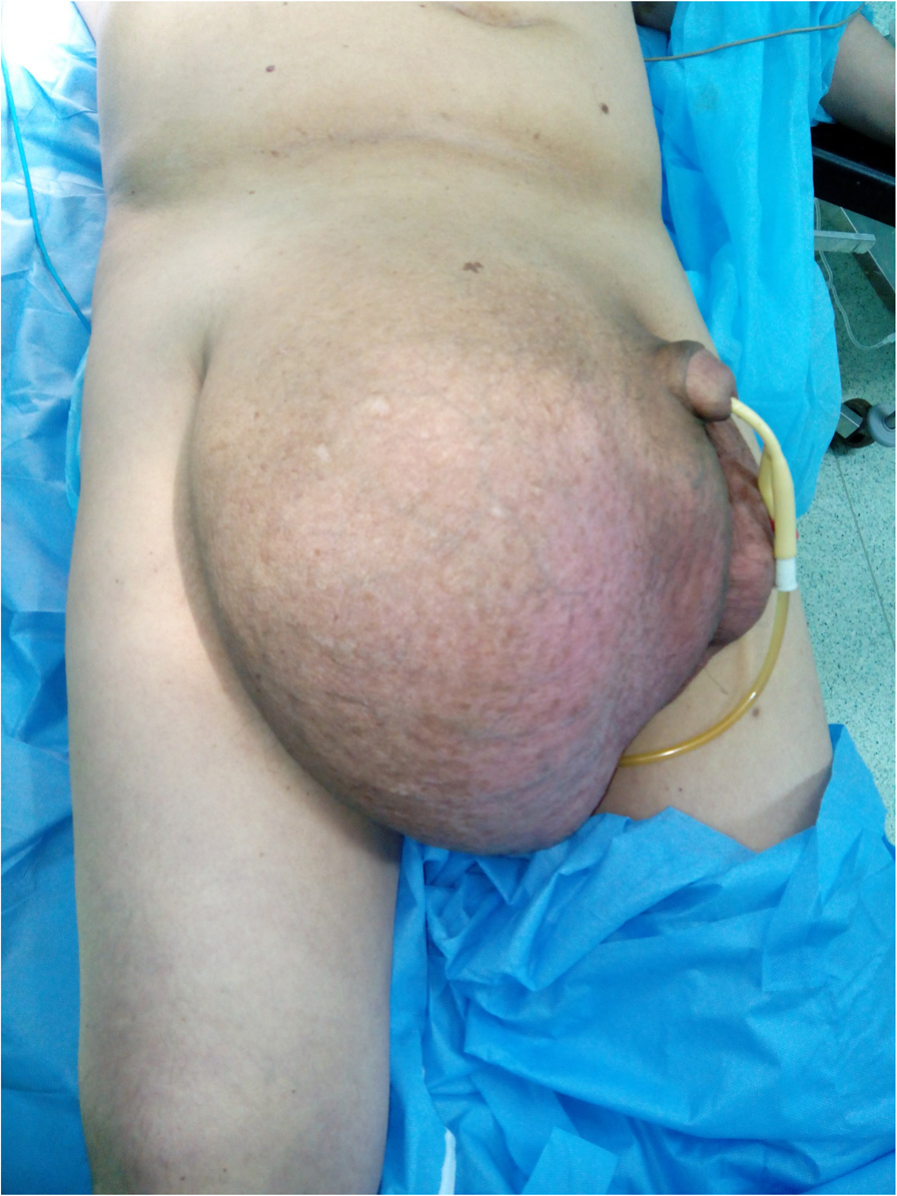
Preoperative photograph of the patient’s giant right-sided inguinoscrotal hernia extending below the midpoint of the inner thigh. The patient is in supine position

**Fig. 2:**
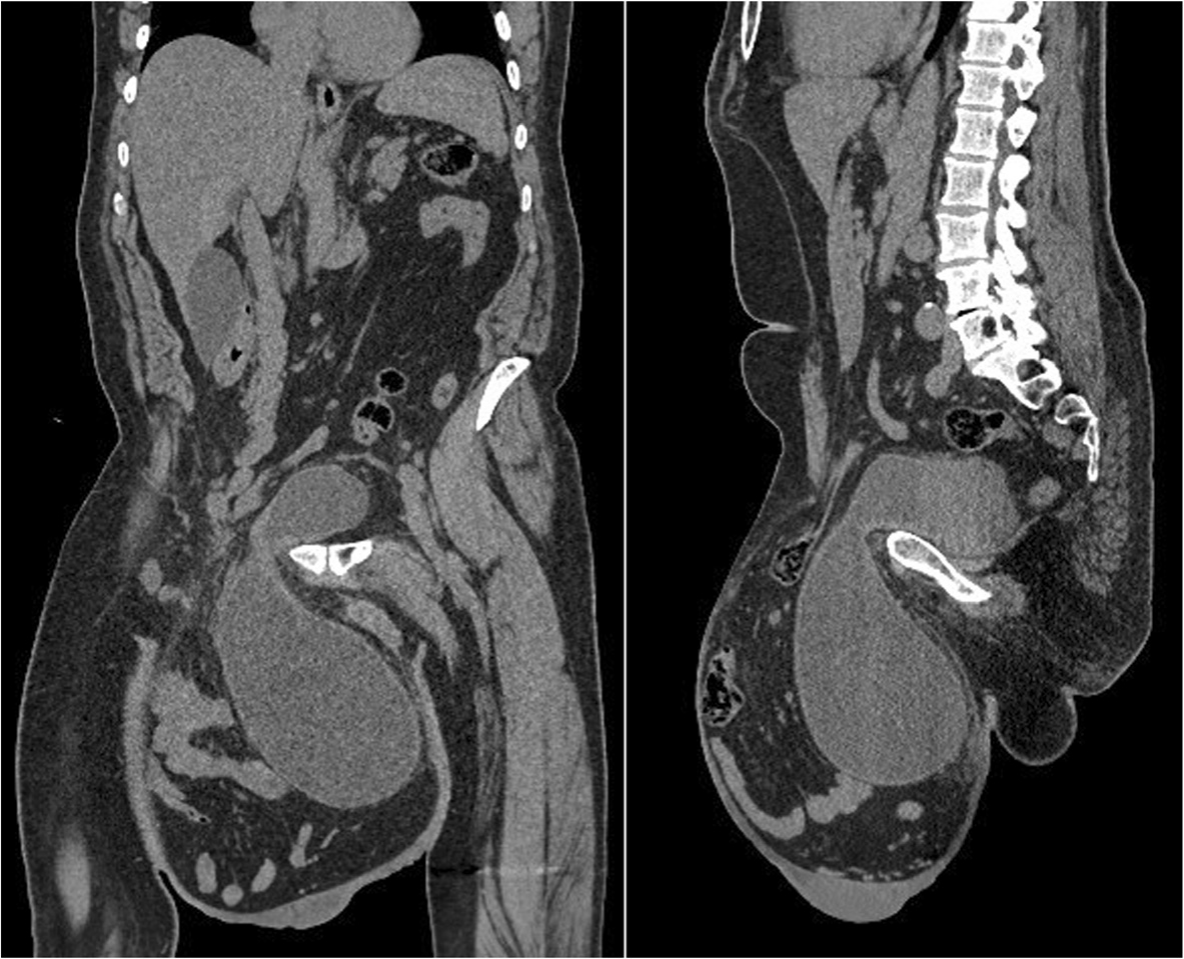
Computed tomography findings. Coronal and sagittal sections show urinary bladder and intestinal segments incarcerated in the large inguinoscrotal hernia sac

**Fig. 3:**
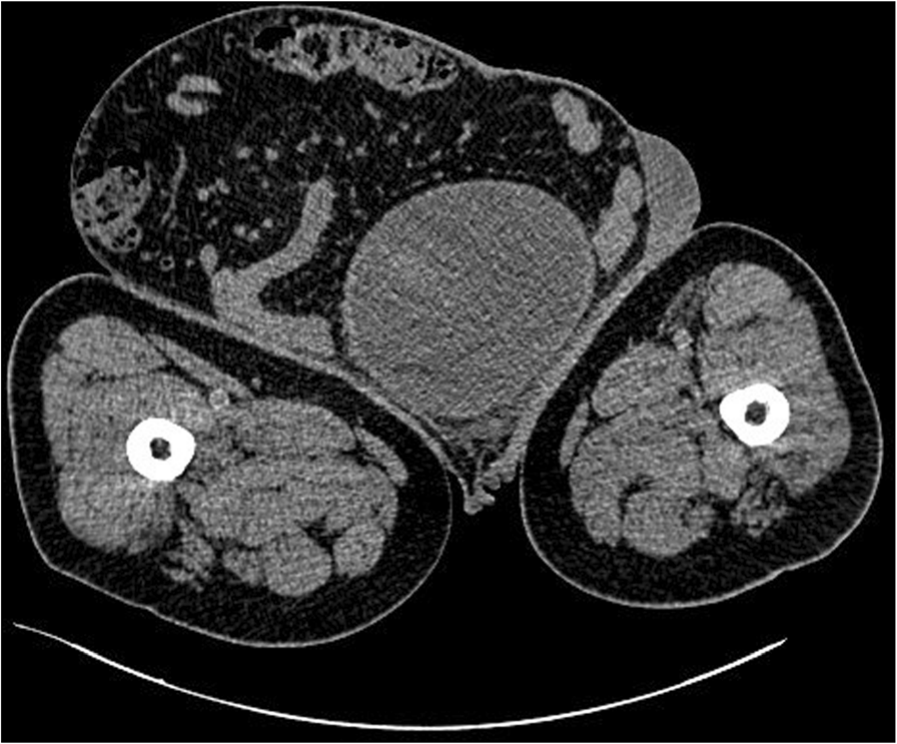
Axial computed tomography demonstrating urinary bladder and bowel loops at the level of the femur

**Fig. 4:**
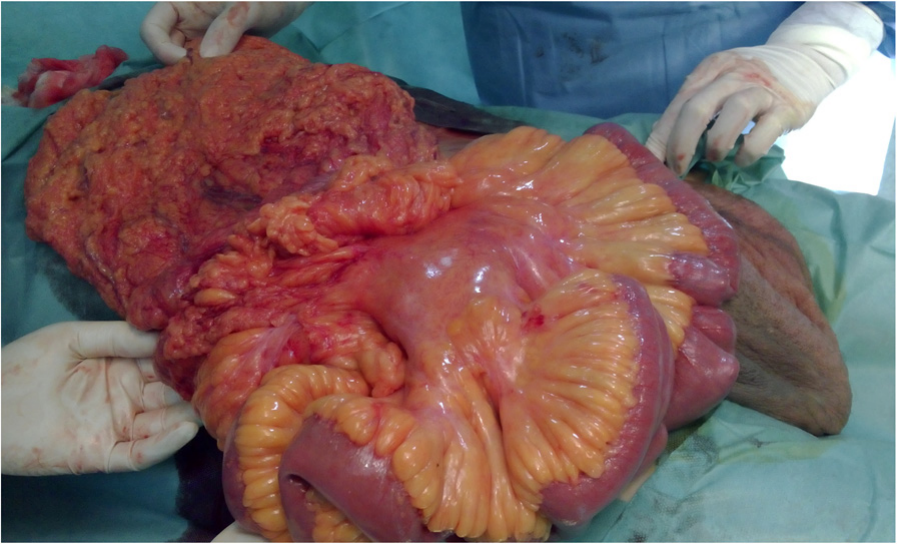
Intraoperative photograph of part of the hernia sac contents

**Fig. 5:**
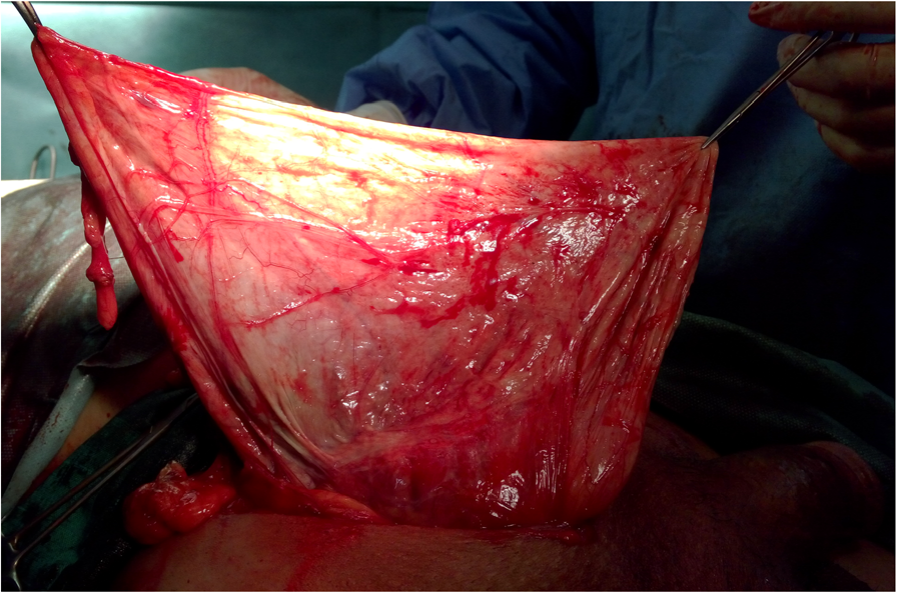
Intraoperative photograph of the patient’s giant hernia sac

A 65-year-old Moroccan man presented with a longer than 12-year history of a right-sided inguinoscrotal hernia, for which he had previously refused any surgical treatment. He reported a large inguinoscrotal swelling that was gradually getting bigger and recently reached below the level of his mid-thigh. He also described episodic abdominal pain and increasingly difficult urination. This development significantly affected his quality of life, with difficulties in walking and sexual activity, prompting him to finally accept surgical management. He had no history of chronic cough, constipation, or previous surgery. Clinical examination revealed a very large, irreducible, no cough impulse, and non-tender right inguinoscrotal hernia. The hernia mass was about 30 cm in size and extended to the midpoint of the inner thigh. The scrotal skin was thickened, but there were no signs of inflammation, excoriation, or ulceration (Fig. 1). Computed tomography demonstrated a voluminous hernia sac containing small and large bowel loops, greater omentum, and urinary bladder (Figs. 2 and 3). The patient underwent surgery while under general anesthesia. A Foley catheter was inserted before the incision was made. The hernia was approached through a right inguinal incision. The hernia sac was dissected and carefully separated from cord structures. The sac contained most of the small bowel with its mesentery, cecum with ascending colon, sigmoid colon, and greater omentum and urinary bladder (Figs. 4 and 5). There was no evidence of ischemia or any adhesions. Manual reduction of the hernia contents into the abdominal cavity was achieved with difficulty after widening of a deep inguinal ring and a partial omentectomy, but without any bowel resection. Also, the urinary bladder was easily repositioned to its pelvic position without injury, and the majority of the hernia sac was resected. Next, the previously enlarged internal inguinal ring was repaired with interrupted non-absorbable monofilament sutures, and the hernia was treated with polypropylene mesh using the Lichtenstein tension-free technique. Last, the patient’s hemostasis was checked and a suction drain was placed on the mesh and in the scrotum. The patient was extubated during the immediate postoperative period and did not require any mechanical ventilatory support. Mild difficulty on respiration was seen and was successfully managed during the first 3 postoperative days in the intensive care unit. On the sixth day after surgery, the drain was removed and the patient was discharged. At his 6-month follow-up examination, the hernia repair was intact without signs of recurrence, scrotal hydrocele, or seroma.

## Discussion

In high-income countries, the diagnosis of inguinal hernia is usually made early, when the patient notices the development of swelling or groin pain. Given the potential hernia strangulation, surgical correction is often carried out without delay. Consequently, giant inguinoscrotal hernias have become extremely rare and are currently seen in clinical practice only after years or even decades of self-neglect [2, 3]. In addition to the classical complications of inguinal hernias, the massive size of giant hernias often causes difficulty in walking, sitting, or lying down, with mobility dramatically restricted. The penis may be buried inside the scrotum causing dribbling of urine over the scrotal skin, which is already congested by lymphatic and venous edema, leading to excoriation, ulceration, and secondary infection [4, 5]. Patients may also complain of difficulty in voiding and recurrent urinary tract infections, especially when the bladder is contained within the hernia sac. These specific problems severely impair the patient’s quality of life, with considerable psychological and social impact [6]. In our patient, the small bowel with its mesentery, cecum with ascending colon, sigmoid colon, and greater omentum were found in a right-sided inguinoscrotal hernia, together with urinary bladder. Such a case has not been reported in the medical literature to date, to the best of our knowledge.

Repair of a giant inguinal hernia is a real challenge, even for experienced surgeons. Abrupt and forced reduction of massive contents of the hernia sac into the limited space of the peritoneal cavity leads to a sudden increase of intraabdominal and intrathoracic pressures. This can cause abdominal compartment syndrome, resulting in compromised respiratory and cardiac function due to splinting of the diaphragm and reduction of venous return [7]. This syndrome is associated with a worsening of morbidity and mortality rates. In addition, reintroduction of the intestine into the abdomen may also cause intestinal obstruction, wound dehiscence, and hernia recurrence.

Surgical options for combating this loss of domain may be divided into either distending the abdominal wall to increase the abdominal space or debulking the abdominal contents. The enlargement of the peritoneal cavity by progressive preoperative pneumoperitoneum has been highly recommended in the past [8, 9], but usually it causes expansion of the thin hernia sac rather than the contracted abdominal cavity, requires prolonged preoperative hospitalization, and fails several times. However, lengthening of the abdominal wall has been described using prosthetic mesh and a scrotal skin flap after creating an anterior abdominal wall defect [10]. Later, a modification was proposed by using the hernia sac as a peritoneal flap [11]. Similarly, a tensor fasciae latae musculocutaneous flap was alternatively used to cover mesh at an anterior abdominal wall defect, and component separation techniques have also been described [12, 13]. As for debulking of the hernia contents, this approach, which includes resection of usually the colon, small intestine, or greater omentum, has also been reported with some success, but it is associated with a greater risk of prosthetic infection or anastomotic leakage [9, 14].

In our patient, none of these techniques were mandatory. Therefore, in spite of massive and specific content, we performed a forced reduction through only an enlarged deep inguinal ring. No debulking or abdominal enlargement procedure had to be performed, apart from a partial omentectomy. Operative repair was achieved through a standard inguinal incision with polypropylene mesh using the Lichtenstein tension-free technique. The mesh, positioned over the posterior wall of the inguinal canal, helps to strengthen the weakened abdominal wall, thus affording significant reduction of the risk of hernia recurrence. According to recent medical literature, current guidelines suggest the Lichtenstein technique as the preferred surgical option for repair of giant inguinal hernias [4, 15]. Moreover, close postoperative monitoring of intraabdominal and intrathoracic pressure is essential in the management of these patients. Our patient developed mild respiratory distress after surgery but was successfully managed in the intensive care unit. Otherwise, a compression bandage with adequate drainage should be implemented to prevent the development of a large scrotal hematoma. Additionally, most authors agree that the scrotal skin should be left redundant, as it retracts due to the dartos muscle contractions and acts as a safety net to allow the contents temporarily back into the scrotum if the patient develops postoperative respiratory compromise [7, 13].

## Conclusions

Giant inguinal hernias are uncommonly encountered in modern surgical practice. However, surgical repair is challenging because of the potentially fatal complications related to increased intraabdominal pressure and the risk for recurrence, which is much higher than in other inguinal hernias. Therefore, adequate preoperative preparation, combined with intensive postoperative monitoring, is essential to obtaining good outcomes. The Lichtenstein tension-free technique seems to be the best surgical procedure for both the patient and the operating surgeon. It should be used whenever possible. Informed consent from the patient is needed to cover all possible surgical options, because the final decision regarding surgery is generally made intraoperatively.

## Consent

Written informed consent was obtained from the patient for publication of this case report and any accompanying images. A copy of the written consent is available for review by the Editor-in-Chief of this journal.

## References

[1] Hodgkinson DJ, McIlrath DC. Scrotal reconstruction for giant inguinal hernias. Surg Clin North Am. 1984;64:307–13. 10.1016/s0039-6109(16)43287-1 6233734

[2] Panagiotakis GI, Spyridakis KG, Chatziioannou MN, Kontopodis NG, Kandylakis SE. Repair of an inguinoscrotal hernia containing the urinary bladder: a case report. J Med Case Rep. 2012;6:90. 10.1186/1752-1947-6-90 PMC3338380 22448739

[3] Patsas A, Tsiaousis P, Papaziogas B, Koutelidakis I, Goula C, Atmatzidis K. Repair of a giant inguinoscrotal hernia. Hernia. 2010;14:305–7. 10.1007/s10029-009-0533-y 19590814

[4] Dinesh HN, Kumar CDJ, Shreyas N. Giant inguinoscrotal hernia repaired by Lichtenstein’s technique without loss of domain - a case report. J Clin Diagn Res. 2014;8:ND07–8. 10.7860/JCDR/2014/9662.4882 PMC4225935 25386483

[5] Karthikeyan VS, Sistla SC, Ram D, Ali SM, Rajkumar N. Giant inguinoscrotal hernia—report of a rare case with literature review. Int Surg. 2014;99:560–4. 10.9738/INTSURG-D-13-00083.1 PMC4253924 25216421

[6] Petersen LF, Luu MB. Giant inguinal scrotal hernia containing the sigmoid colon. Am Surg. 2014;80:e185–6. 24987878

[7] Coetzee E, Price C, Boutall A. Simple repair of a giant inguinoscrotal hernia. Int J Surg Case Rep. 2011;2:32–5. 10.1016/j.ijscr.2010.11.005 PMC3199728 22096682

[8] Piskin T, Aydin C, Barut B, Dirican A, Kayaalp C. Preoperative progressive pneumoperitoneum for giant inguinal hernias. Ann Saudi Med. 2010;30:317–20. 10.4103/0256-4947.65268 PMC2931785 20622351

[9] Vasiliadis K, Knaebel HP, Djakovic N, Nyarangi-Dix J, Schmidt J, Büchler M. Challenging surgical management of a giant inguinoscrotal hernia: report of a case. Surg Today. 2010;40:684–7. 10.1007/s00595-009-4125-3 20582525

[10] Merrett N, Biankin A. Giant inguinal hernia containing right colon repaired using the PROLENE hernia system. ANZ J Surg. 2009;79:92–3. 10.1111/j.1445-2197.2008.04817.x 19183397

[11] El-Dessouki NI. Preperitoneal mesh hernioplasty in giant inguinoscrotal hernias: a new technique with dual benefit in repair and abdominal rooming. Hernia. 2001;5:177–81. 10.1007/s10029-001-0030-4 12003044

[12] Hamad A, Marimuthu K, Mothe B, Hanafy M. Repair of massive inguinal hernia with loss of abdominal domain using laparoscopic component separation technique. J Surg Case Rep. 2013;2013(3):rjt008. 10.1093/jscr/rjt008 PMC3635196 24964420

[13] Mehendal FV, Taams KO, Kingsnorth AN. Repair of a giant inguinoscrotal hernia. Br J Plast Surg. 2000;53:525–9. 10.1054/bjps.2000.3383 10927688

[14] Trakarnsagna A, Chinswangwatanakul V, Methasate A, Swangsri J, Phalanusitthepha C, Parakonthun T, et al. Giant inguinal hernia: report of a case and reviews of surgical techniques. Int J Surg Case Rep. 2014;5:868–72. 10.1016/j.ijscr.2014.10.042 PMC4245684 25462054

[15] Bierca J, Kosim A, Kołodziejczak M, Zmora J, Kultys E. Effectiveness of Lichtenstein repairs in planned treatment of giant inguinal hernia - own experience. Wideochir Inne Tech Maloinwazyjne. 2013;8:36–42. 10.5114/wiitm.2011.30971 PMC3627151 23630552

